# Editorial: Immunometabolism in autoimmune and autoinflammatory disorders

**DOI:** 10.3389/fimmu.2023.1285720

**Published:** 2023-11-01

**Authors:** Fang Zheng, Geert Raes, Shemin Lu, Qian Chen

**Affiliations:** ^1^ Department of Biochemistry and Molecular Biology, The Key Laboratory of Environment and Genes Related to Disease of Ministry of Education, Health Science Center, Xi’an Jiaotong University, Xi’an, China; ^2^ Research Group of Cellular and Molecular Immunology, Vrije Universiteit Brussel, Brussels, Belgium; ^3^ Myeloid Cell Immunology Lab, Vlaams Instituut voor Biotechnologie (VIB) Center for Inflammation Research, Brussels, Belgium; ^4^ Department of Orthopaedics, Alpert Medical School of Brown University, Rhode Island Hospital, Providence, RI, United States

**Keywords:** immunometabolism, autoimmune disorders, autoinflammatory disorders, immune cells, immunometabolism-associated diseases

Over the past years, immunometabolism and its role in autoimmune and autoinflammatory disorders have become a growing Research Topic. Immunometabolism refers to studying how cellular metabolism impacts immune cell functions and responses. Multiple factors are contributing to the immunological response of the human immune system. Immune cell signaling and differentiation processes control a number of metabolic pathways in a way that determines the fate of immune cells. Pathogenesis of autoimmune disorders is influenced by metabolic alterations of immune cells. Some immune diseases, such as rheumatoid arthritis, systemic lupus erythematosus, and multiple sclerosis, have been evidenced to exacerbate in response to metabolic alterations and dysregulation of immune cells, including T cells, B cells, and macrophages, contributing to the pathogenesis of the disease. On the other side, alteration of metabolic pathways in innate immune cells, such as monocytes, neutrophils, and natural killer cells, can contribute to excessive pro-inflammatory responses. Certain autoinflammatory disorders, such as Schnitzler syndrome and TNF receptor-associated periodic syndrome, are attributed to an alteration of fatty acid metabolism and mitochondrial dysfunction. However, recognizing metabolic alterations as key contributors to disease pathogenesis, like the immunometabolism regulators and effectors, could provide hints for novel therapeutic targets to modulate metabolic programming and immune cell responses in autoimmune and autoinflammatory disorders.

In this Research Topic, we aim to discuss the current challenges in immunometabolism, particularly those attributed to autoimmune and autoinflammatory disorders, as well as the current gaps, limitations, and prospects for developing novel therapeutic targets against autoinflammatory disorders. The ultimate goal of this research topic is to provide an advanced comprehension of the immuno-metabolic mechanisms interfering with autoinflammatory disorders and explore the innovative ways of regulating the endogenous metabolites with anti-inflammatory effects aiming to restrain the prevalence of autoimmune and autoinflammatory disorders via specific targeting of the various metabolic events.

Rheumatoid vasculitis (RV) is an uncommon, extra-articular manifestation in patients with long-standing seropositive rheumatoid arthritis (RA). The mean duration between the diagnosis of RA and the onset of vasculitis is typically 10–14 years. In their research entitled: “*Peripheral ulcerative keratitis, nodular episcleritis, and pulmonary nodules as the initial signs of rheumatic arthritis: a case report*”, Wang et al. have described a rare case of rapidly deteriorated bilateral peripheral ulcerative keratitis (PUK) accompanied by nodular episcleritis and pulmonary nodules in a same patient with RA but without joint involvement. The patient exhibited various symptoms, including crescent-shaped marginal corneal ulcers in both eyes, nodular episcleritis in the right eye, and vasculitis, confirmed by conjunctival biopsy, with positive rheumatoid factor and anti-cyclic citrullinated protein antibody. Despite using topical and systematic corticosteroids and other immunosuppressive medications, the ocular condition was not relieved until the initiation of the infliximab therapy. However, PUK recurred after discontinuing the infliximab therapy. This case highlighted the RV as a prospective initial sign of RA. It also suggests that infliximab could be beneficial in preventing further progression of RA-related PUK in refractory cases.

Osteoarthritis (OA) is a prevalent chronic degenerative joint disorder that manifests a wide variety of symptoms including joint pain, stiffness, hypertrophy, deformity, and restricted movement. Articular cartilage degradation and concomitant adaptive osteogenesis are characteristics of OA. Liao et al., have analyzed the existing literature on the role of autophagy in OA to identify global research trends and hotspots in their bibliometric study entitled: “*Knowledge mapping of autophagy in osteoarthritis from 2004 to 2022: a bibliometric analysis*”. Autophagy, the cellular degradation and recycling process of cartilage, is essential in maintaining cellular homeostasis by mediating both cell survival and apoptosis. Several researchers have focused on understanding the mechanism underlying autophagy and OA, including AMPK, macrophages, TGF-β1 inflammatory response, stress, and mitophagy, according to the study. The emerging research trends focus on investigating the connection between autophagy, apoptosis, and senescence and exploring promising drug candidates such as TXC and green tea extract. The researchers support the trend of developing targeted drugs that enhance or restore autophagic activity as a novel strategy for treating OA. The study thoroughly evaluates the current literature on autophagy in OA and proposes prospective treatment options.

Perivascular adipose tissues (PVAT) were examined by Shi et al. in their work entitled: “*Perivascular Adipose Tissue Promotes Vascular Dysfunction in Murine Lupus*” which investigated the role of PVAT in systemic lupus erythematosus (SLE) and how it impacts cardiovascular disease (CVD) in lupus patients. Using lupus mouse models, the researchers investigated the phenotype and function of PVAT as well as the mechanisms relating PVAT to vascular dysfunction in SLE lupus disease. According to the findings, the PVAT from lupus mice exhibits dysfunctional and inflamed characteristics, including impaired endothelium-dependent relaxation of the thoracic aorta, phenotypic switching, immune cell infiltration, and adventitial hyperplasia. The outcomes suggest the involvement of PVAT in vascular disease in lupus, providing new insights into the pathophysiology of cardiovascular complications in lupus and implying that targeting PVAT might be a potential therapeutic approach in lupus.

Luo et al. comprehensively reviewed the relationship between cholesterol metabolism and psoriasis. Psoriasis is a prevalent chronic autoinflammatory skin disease widely recognized as a systemic inflammatory rather than a merely cutaneous disease. Their review, entitled: “*Crosstalk Between Cholesterol Metabolism and Psoriatic Inflammation*”, emphasized the strong association between cholesterol and lipid metabolism alterations and the onset of psoriasis. They also discussed the effects of cholesterol metabolites and cytokines on keratinocytes, immune response, and inflammation in psoriasis. This research fills a gap in the literature by thoroughly examining the connection between cholesterol metabolism and psoriasis, offering novel insights and promising therapeutic targets for further investigation.

In conclusion, we believe this Research Topic has provided a timely opportunity for researchers to showcase their work and explore the interplay between immunometabolism and immunometabolism-associated diseases, notably autoimmune and autoinflammatory disorders. As such, it has opened the way for innovative therapeutic approaches.

## Author contributions

FZ: Writing – original draft, Writing – review & editing. GR: Writing – review & editing. SL: Writing – review & editing. QC: Writing – review & editing.

